# Analysis of Nasal Foreign Bodies in South Korea: Over 10-Year Experience

**DOI:** 10.3390/diagnostics12081810

**Published:** 2022-07-28

**Authors:** Hahn Jin Jung, Sun Wook Kim, Joong Seob Lee, Hyo Geun Choi, Jee Hye Wee

**Affiliations:** 1 Department of Otorhinolaryngology-Head & Neck Surgery, Chungbuk National University Hospital, Chungbuk National University College of Medicine, Cheongju 28644, Korea; hahnjin2@naver.com; 2 Department of Otorhinolaryngology-Head & Neck Surgery, Hallym University Sacred Heart Hospital, Hallym University College of Medicine, Anyang 14068, Korea; hallymin@hallym.or.kr (S.W.K.); apniosio@naver.com (J.S.L.); pupen@naver.com (H.G.C.)

**Keywords:** foreign bodies, nasal cavity, batteries, complications, nasal septal perforation

## Abstract

Foreign bodies (FBs) in the nasal cavity are commonly encountered in otorhinolaryngology. This retrospective study was conducted between January 2009 and December 2020. The nasal FB frequencies were investigated according to age and sex. The type, shape, and location of the FBs, onset, and clinical features were obtained. In a total of 1228 cases, the average age was 3.9 ± 5.7 years. We found a higher incidence in patients 2–4 years old. The most frequent nasal FBs were beads (24.0%), toys/plastics (17.8%), and beans/corn (15.8%). However, there were some differences in the nasal FB types according to the age group. There was no difference in the location of the nasal FBs according to age group, but nasal FBs were found more frequently in the right nasal cavity in the 1–3-years-old groups. Most patients (97.5%) visited the hospital the same day when the nasal FB insertion was suspected, and most were asymptomatic. After the removal of the nasal FBs, most patients were free of complications. Of the nine cases involving button batteries, septal perforation was observed in two patients who visited the hospital one or two days after the FB insertion. There were specific age-based characteristics of the nasal FBs that may serve as a basis for specific precautions.

## 1. Introduction

Foreign bodies (FBs) of the nose are frequently encountered in otorhinolaryngology clinics, particularly in children. In a nationwide epidemiological report of pediatric FB injury-related emergency department visits in South Korea, the most common anatomical site of FB injury was the nose (36.0%) [1]. The most common age of presentation was reported to be between 2 and 4 years [2]. Although most nasal FBs do not cause any problems because the insertion of the FB is witnessed by other people and is relatively easily removed in the clinic, an inserted nasal FB can damage the nasal mucosa/cartilage and result in potentially serious complications. In a European survey of FB injuries, the rates of complication and hospitalization associated with a nasal FB were reported to be 8.6 and 7.5%, respectively [3].

Although many studies on nasal FBs have focused on children [4,5,6,7], it is important to evaluate the frequency and clinical characteristics of nasal FBs in other age groups. To the best of our knowledge, only a few studies have reported the frequency, type, and location of nasal FBs, not just in children, according to age. This study aimed to document the prevalence of nasal FBs according to the age groups in a large number of patients over 10 years from a single tertiary care center in South Korea. Moreover, we investigated other clinical characteristics, such as the accompanying symptoms, the period from the suspected time of the nasal FBs insertion to the hospital visit, complications, and the type and location of the nasal FBs, according to the age groups.

## 2. Materials and Methods

This study was approved by the Hallym University Sacred Heart Hospital institutional review board (2019-11-018). Retrospective reviews of the medical records were performed for all patients who visited the otorhinolaryngology outpatient clinic or emergency unit of Hallym University Sacred Heart Hospital on suspicion of nasal FBs with the diagnostic code of ICD-10: T170-T172 from January 2009 to December 2020.

All patients were examined using rigid endoscopy in the outpatient clinic to evaluate the presence of a FB in the nasal cavity or nasopharynx. In some cases, in which nasal FBs could not be identified, radiography was used for further evaluation. Data on age, sex, onset, type and location of FBs, symptoms, endoscopic findings, and complications were collected.

The age groups were divided in 1-year intervals for preschool patients (<1, 1–2, 2–3, …, 6–7 years), elementary (7–13 years) and middle/high school (13–19 years) students for school-age patients, and under and over 60 years for adults. The time from suspected nasal FB insertion to visiting the hospital was classified as the same day or number of days. As most of them were children, parents sometimes visited when they directly witnessed the insertion of a nasal FB, but in many cases, they did not know the exact time of insertion. Nasal FBs were categorized as organic vs. inorganic, and as round vs. long vs. irregular. The specific types of nasal FBs were beads, toys/plastics, beans/corn, cotton/toilet paper, paper/sticker, snack, fruit/vegetable, silicon/rubber, clay, button, battery, magnets, etc. The anatomic locations of the nasal FBs were classified based on the detected region. The location was described as the right, left, or both of nasal cavity.

Statistical analyses were performed using SPSS 22.0 (IBM, Armonk, NY, USA). Chi-square analysis was used to evaluate the differences in categorical variables, such as types, locations, and onset of suspected FBs, according to age group. Statistical significance was set at *p* < 0.05.

## 3. Results

**Figure 1:**
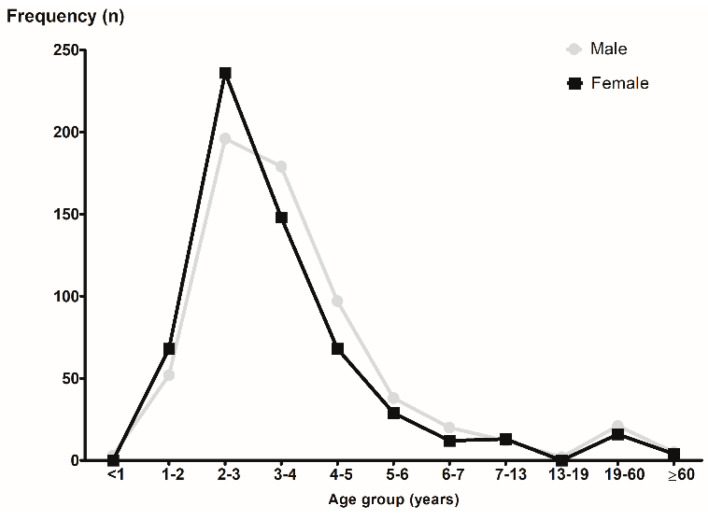
The frequencies of nasal foreign body according to the age group and sex.

**Figure 2:**
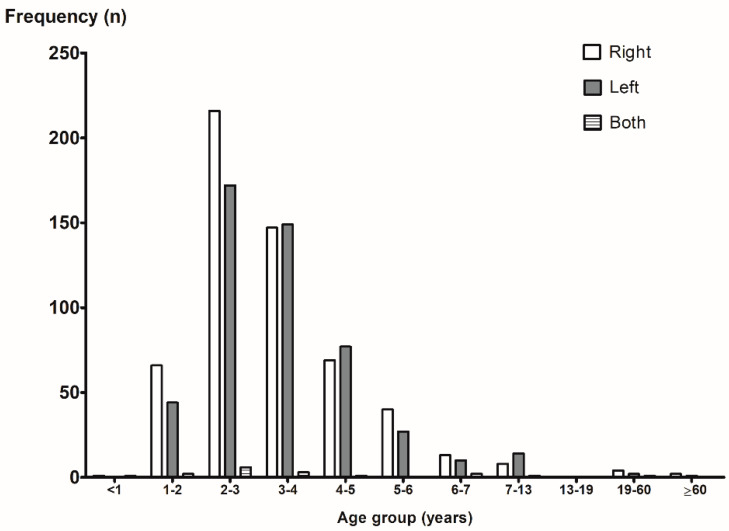
The frequencies of nasal foreign body according to the age group and location.

A total of 1228 patients with a mean age of 3.9 ± 5.7 years (range: 0.5–78.5 years) were included. There were 632 males (51.5%) and 596 females (48.5%). There was no significant difference in the mean age between males (4.2 years) and females (3.7 years) (*p* = 0.142). Figure 1 shows the frequencies of the nasal FBs according to age and sex. The frequency of the nasal FBs was markedly higher in the group of patients aged 2–4 years and lower in the group of patients aged 13–19 years (*p* for trend = 0.008). There were more girls than boys only in the 1–3-years-old groups. There was no difference in the side where the FB was located (right, 52.4%; left, 46.0%; both, 1.6%). However, in the 1–3-years-old groups, nasal FBs were found more frequently in the right nasal cavity (Figure 2).

**Figure 3:**
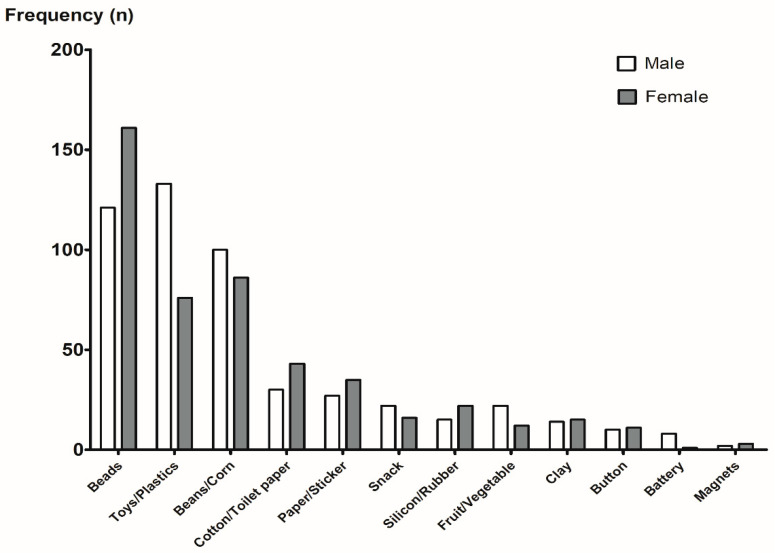
The types of nasal foreign body according to the sex.

Among them, 51 patients (4.2%) were suspected of having a nasal FB insertion, but it was not found even after radiography was performed. According to the age group, a nasal FB was not found in 2.9% (13/444)–6.3% (11/175), 41.7% (5/12), and 25.5% (1/4) of the cases in young children aged 1–7 years, in the 19–60 years adult group, and in the over 60-years-old group, respectively. However, nasal FBs were always found when suspected in the 7–13-years-old group. Of the 1177 patients, the most common nasal FBs were beads (24.0%), followed by toys/plastics (17.8%) and beans/corn (15.8%) (Table 1). We observed differences in the types of nasal FBs according to the sex (Figure 3) and age group (Table 2). The most common nasal FBs were beads, followed by beans/corn and toys/plastic in the female group, and toys/plastic, followed by beads and beans/corn in the male group (Figure 3). The most common nasal FBs in the 1–3-years-old groups were beans/corn, followed by beads and toys/plastics. In contrast, in the 3–5-years-old groups, the most common nasal FBs were beads, followed by toys/plastics and beans/corn. In the 5–7-years-old groups, the most common nasal FBs were beads, followed by toys/plastics and cotton/toilet paper. In the adult group, the most common nasal FB was cotton/toilet paper (Table 2).

**Figure 4:**
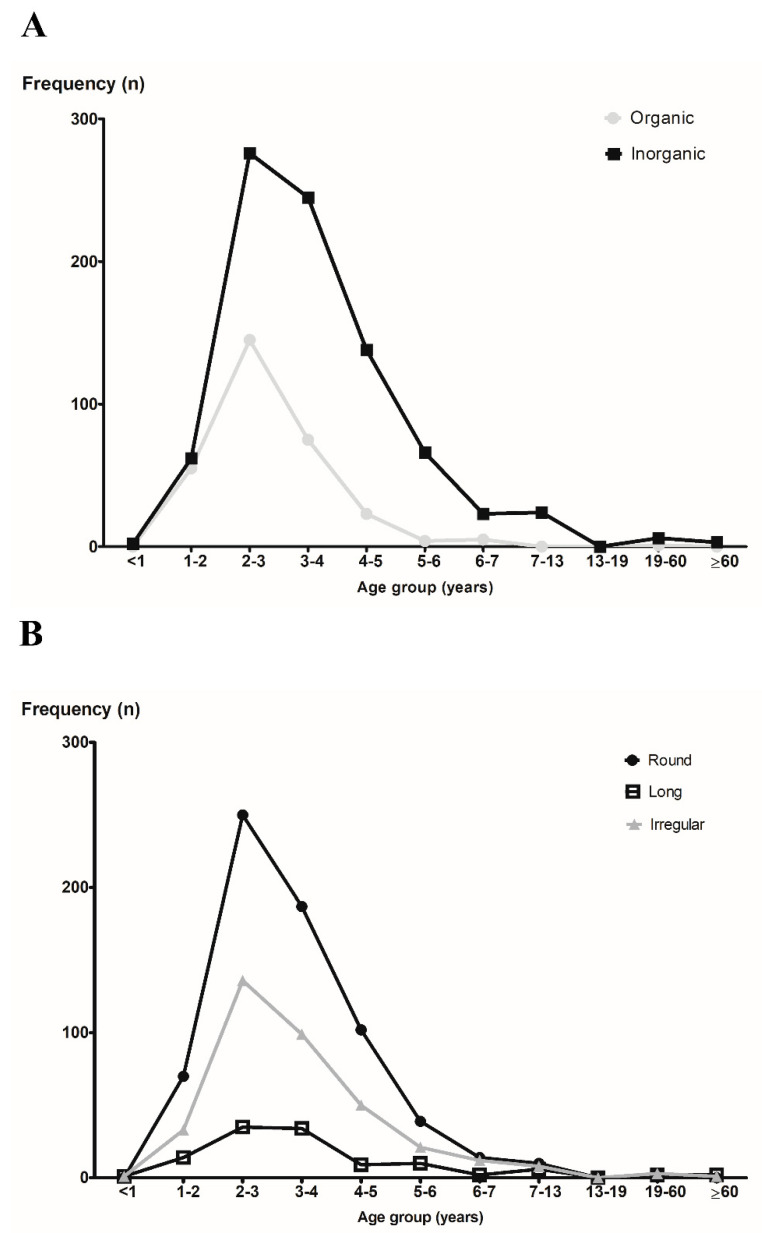
The frequencies of nasal foreign body according to the age group. (**A**) Organic vs. inorganic and (**B**) round vs. long vs. irregular.

Excluding cases (n = 24) where it was difficult to know the exact type of FB, 845 (73.3%) were inorganic and 308 (26.7%) were organic (Table 3). The most common shape of the nasal FBs was round-shaped objects (58.5%), such as beads, beans, fruit seeds, and nuts. The second most common shape of the nasal FBs was irregular objects (31.5%), such as toys, cotton, paper, and rubber (Table 3). These results showed the same pattern across all age groups (Figure 4A,B).

Most patients (n = 1197, 97.5%) visited the hospital on the same day when the nasal FB insertion was suspected, 21 patients (1.7%) visited the hospital 1 day later, and 6 patients (0.5%) visited the hospital after 2 days or more (range: 2–14 days). Four patients (0.3%) did not know how many days they suspected having a nasal FB insertion. When visiting the hospital on the same day, most of them had no symptoms, but when more than one day passed, they presented with symptoms such as rhinorrhea and nasal obstruction (Table 4). There were not many cases of abnormal endoscopic findings after the nasal FBs were removed (mucosal erosion in seven cases, bloody crust in five cases, mucosal edema in four cases, pus discharge in four cases, and synechia in one case).

After the removal of the nasal FBs, most patients were free of complications; however, complications occurred in four patients: septal perforation (n = 2) and sinusitis (n = 2). Two patients with complications of septal perforation had the FB (battery) removed, who visited the hospital one or two days after the FB insertion. None of the remaining seven patients who underwent a battery insertion in the nasal cavity and visited the hospital on the same day had any complications.

## 4. Discussion

The present study documented the prevalence and clinical features of nasal FBs according to age in a large number of patients over 10 years in South Korea. In addition, we evaluated nasal FBs in all age groups, although nasal FBs is specifically a common problem in the pediatric age group.

We showed the differences in the types of nasal FBs according to the age group. Previous studies have reported the types of FBs commonly found in the nasal cavity, but there have been few reports of differences in the nasal FB types according to the age group. We classified the specific types of nasal FBs into beads, toys/plastics, beans/corn, cotton/toilet paper, paper/sticker, snack, fruit/vegetable, silicon/rubber, clay, buttons, batteries, magnets, and others. Beads were the most common type of nasal FB in children of all ages except those 1–3 years. However, in the 1–3-years-old group, beans/corn were the most common type of nasal FB. In a study in which more than 55% of the cases involved children under 4 years of age, organic seeds were also commonly retrieved [8]. Organic FBs, such as beans and corn, tend to swell and are usually more difficult to remove. In a European survey of FB injuries, the most common FBs associated with complications and hospitalization were spherically shaped objects, such as nuts, seeds, berries, peas, corn, and beans [3].

When we divided the nasal FBs into organic and inorganic materials, the most common nasal FBs were inorganic compounds, accounting for 73.3% of the extracted objects. This finding was consistent with those of previous reports [5,7,9]. The most common shape of nasal FBs was round-shaped objects (58.5%), such as beads, beans, fruit seeds, and nuts. These results did not differ across the age groups. If the shape of the nasal FB is round, it is more likely to go deep into the nasal cavity during the removal process. Therefore, the use of forceps is not recommended; in contrast, the use of a hook or suction catheter is recommended. A previous work reported that the success of nasal FB removal may depend on the shape of the FBs [7]. In addition, we summarized the yearly counts to assess the trends over time (Supplementary Figure S1). Overall, the incidence of nasal FBs showed a trend of increasing and then decreasing over time in the present study, in contrast to the results of a previous study [10]. Among the two common types (beads and beans/corn), beans/corn showed a declining trend. We suppose that steady parental education about the risk of bean or corn aspiration in children is the reason for this decreasing trend. Prevention remains the best treatment, implying strengthened education of caretakers, such as parents and babysitters, on age-appropriate foods and household items.

This study demonstrated a higher frequency (63.7%) of a nasal FB in patients aged 2–4 years, which was in line with previous studies [4,5,9,11]. The incidence of nasal FBs decreased dramatically after the age of 6 years. The predominance of this age group may be associated with growth-related factors around the age of 2–4 years, such as walking well, grasping easily with their fingers, and increasing interest in their own body. This process can cause the placement of FBs in the nasal orifices. In addition, there were more girls than boys only in the 1–3-years-old groups. It may be associated with sex differences in early fine motor development, such as grasping with fingers. Previous studies have suggested that girls have greater fine motor skills than boys at 2 and 3 years of age [12,13]. The most common nasal FBs were cotton/toilet paper in the adult group, although this was a small number. Most cases were not removed after insertion into the nose with cotton or toilet paper because of rhinorrhea or epistaxis.

With regard to sex, 51.5% of our cases were male and 48.5% were female; likewise, other researchers have not found any significant sex difference [7,14,15]. While previous studies have reported that nasal FBs mainly occur in the right nasal cavity, presumably due to right-handed predominance in the population [4,6,7,16], there was no difference in the side where the FB was found (right, 52.4%; left, 46.0%; both, 1.6%) in the present study. However, in the 1–3-years-old groups, nasal FBs were found more frequently in the right nasal cavity.

Radiological assessment is performed if the clinical examination is difficult or inconclusive. In this study, radiography was required in only 54 patients, and no FB was found in 51 patients. Thus, a radiological evaluation is not necessary for most cases of nasal FBs. In the literature, FBs are usually found on the floor of the anterior or middle third of the nasal cavity [3].

In the present study, most of the patients (98.6%) were asymptomatic; although the absence of symptoms was the most common in other studies (34.88–81.9%) [4,11,16,17], the proportion observed in our study was higher. This is probably because most patients (97.5%) presented within 1 day; the percentage of such patients was higher than that reported in previous studies. A study in Malaysia showed that 34.88% of patients were asymptomatic and 27.91% presented within 24 h [17]. A study in Israel reported that 81.9% of children were asymptomatic, and 55 and 70% of those aged < 4 and >4 years were admitted less than 24 h later [4]. This was possible because South Korea has a single mandatory national health insurance system that covers almost the entire population, and access to medical care, including tertiary care, is easy. When more than 1 day had passed, the patients presented symptoms, such as rhinorrhea and nasal obstruction. As the FB insertion was unwitnessed, the diagnosis was made when symptoms occurred.

Batteries are the most common type of FB associated with complications. Any delay can lead to necrosis of the nasal mucosa and septal perforation [18]. However, in this study, septal perforation was observed in only two of the nine patients with a battery nasal FB. Two patients with a complication of septal perforation visited the hospital one or two days after the FB insertion. Some researchers have suggested that the likelihood of septal perforation may be related to the time interval between insertion and removal [19,20].

This study has several limitations. First, this was a retrospective chart review of only one tertiary care center. However, in South Korea, the national health insurance system covers almost the entire population, is cost effective, and has easy access even to tertiary medical institutions. In addition, we reviewed patients for more than 10 years to analyze a large sample size. Second, we could not obtain information on the removal of the nasal FBs. Nasal FBs are usually removed with direct visualization, using forceps, hook, or suction catheters, based on the FB type, to avoid complications during extraction. All nasal FBs were successfully removed in this study and no severe treatment-related complications were observed. Most of our patients did not require sedation for the nasal FB removal, with only four children requiring general anesthesia. This frequency is much lower than that previously reported in other studies [4,7].

## 5. Conclusions

In this over 10-year study of 1228 patients, the frequency of nasal FBs was the highest in children 2–4 years. When grouped according to age, there was a difference in the type of nasal FB but not in the sex and location of the nasal FBs. Nasal FBs are most commonly inorganic and round-shaped objects. Complications from nasal FBs are rare, but a battery lasting more than 1 day is associated with increased septal perforation. There are specific age-based characteristics of nasal FBs that would help provide guidance for the diagnosis and management of patients with nasal FBs.

## Figures and Tables

**Table 1:** Types of foreign bodies in nasal cavity.

Types of Foreign Bodies	Number (Percentage)
Beads	282 (24.0)
Toys/Plastics	209 (17.8)
Beans/Corn	186 (15.8)
Cotton/Toilet paper	73 (6.2)
Paper/Sticker	62 (5.3)
Snack	38 (3.2)
Silicon/Rubber	37 (3.1)
Fruit/Vegetable	34 (2.9)
Clay	29 (2.5)
Button	21 (1.8)
Battery	9 (0.8)
Magnets	5 (0.4)
Others	171 (14.5)

**Table 2:** Top 5 types of nasal foreign bodies according to the age groups.

Age Groups	Top 5 Types of Foreign Bodies (Percentage)
1–2 years	Beans/Corn (34.0%), Beads (17.5%), Toys/Plastics (11.3%), Cotton/Toilet paper (9.3%), Fruit/Vegetable (8.2%)
2–3 years	Beans/Corn (24.5%), Beads (21.6%), Toys/Plastics (16.4%), Cotton/Toilet paper (8.6%), Paper/Sticker (7.5%)
3–4 years	Beads (28.0%), Toys/Plastics (26.2%), Beans/Corn (16.8%), Paper/Sticker (6.1%), Silicon/Rubber (5.0%)
4–5 years	Beads (45.0%), Toys/Plastics (25.5%), Beans/Corn (7.4%), Cotton/Toilet paper (6.0%), Paper/Sticker, Seeds (3.4%)
5–6 years	Beads (42.1%), Toys/Plastics (24.6%), Cotton/Toilet paper (12.3%), Beans/Corn, Paper/Sticker (3.5%)
6–7 years	Beads (30.8%), Toys/Plastics (26.9%), Beans/Corn, Cotton/Toilet paper, Paper/Sticker (3.5%)
7–12 years	Beads (35.0%), Toys/Plastics (25.0%), Cotton/Toilet paper (15.0%), Paper/Sticker, Clay (10.0%)
≥19 years	Cotton/Toilet paper (30.0%), Beads (10.0%), Rubber (10.0%), Fruits/Vegetable (10.0%), Paper/Sticker (10.0%)

**Table 3:** Frequency of nasal foreign bodies according to shape and organic/inorganic matter.

Shape	Organic	Inorganic	Total
Round	276	398	674 (58.5%)
Long	13	102	115 (10.0%)
Irregular	19	345	364 (31.5%)
Total	308 (26.7%)	845 (73.3%)	1153

**Table 4:** Symptoms according to the onset of foreign body insertion.

Symptoms	Onset (Days), Number (Percentage)			
	0	1	≥2	Total
No symptoms	1010 (98.6)	13 (1.3)	1 (0.1)	1024 (83.4)
Foreign body sense	25 (89.3)	2 (7.1)	1 (3.6)	28 (2.3)
Pain	24 (92.3)	1 (3.8)	1 (3.8)	26 (2.1)
Epistaxis	21 (91.3)	0 (0.0)	2 (8.7)	23 (1.9)
Rhinorrhea	12 (66.7)	4 (22.2)	2 (11.1)	18 (1.5)
Cough	10 (90.9)	0 (0.0)	1 (9.1)	11 (0.9)
Nasal obstruction	7 (70.0)	1 (10.0)	2 (20.0)	10 (0.8)
Hoarseness	1 (100.0)	0 (0.0)	0 (0.0)	1 (0.1)
Foul odor	0 (0.0)	1 (100.0)	0 (0.0)	1 (0.1)

## Data Availability

Not applicable.

## Supplementary Materials

The following supporting information can be downloaded at: https://www.mdpi.com/article/10.3390/diagnostics12081810/s1, Figure S1: The frequencies over time of nasal foreign bodies.
