# Supplementary figures and images for: Analysis of Nasal Foreign Bodies in South Korea: Over 10-Year Experience
Source: Diagnostics (Basel). 2022 Jul 28;12(8):1810. doi: 10.3390/diagnostics12081810 (PMC9406659; doi:10.3390/diagnostics12081810)

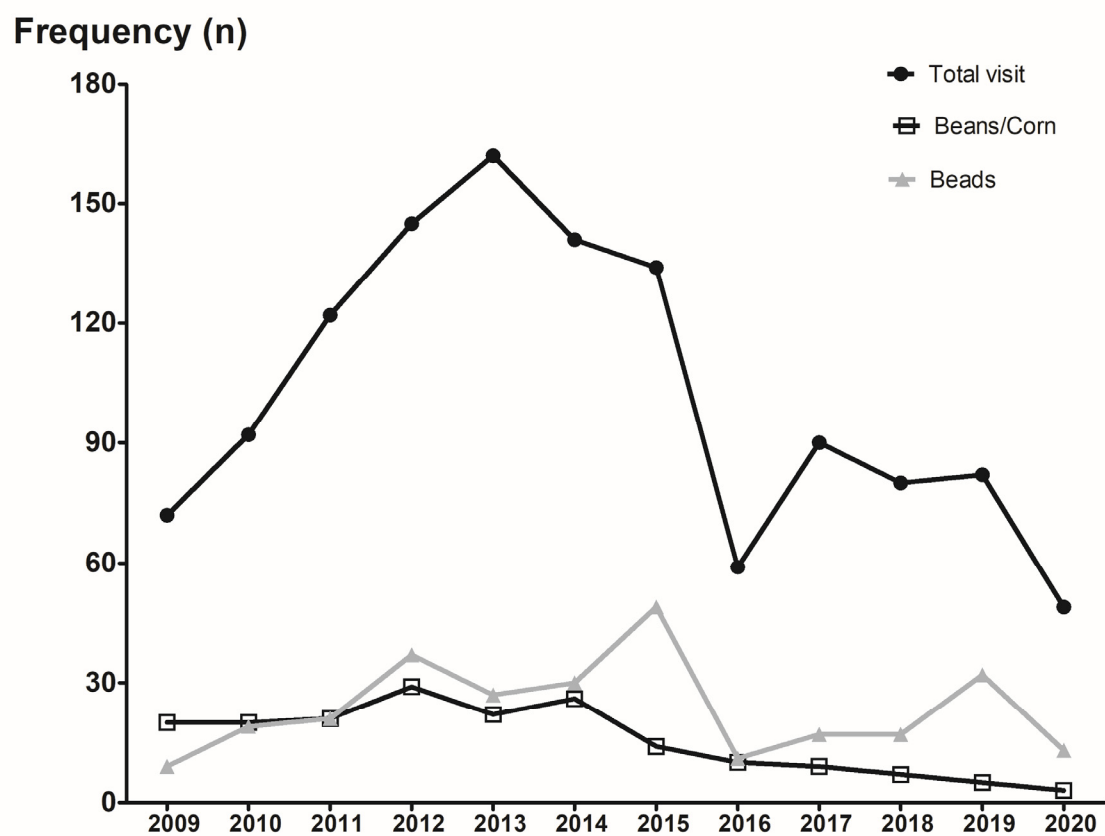

Figure S1. The frequencies over time of nasal foreign bodies.

Supplement: Supplementary file 1 [file diagnostics-12-01810-s001.zip › diagnostics-1827942-supplementary.pdf]
